# Clinical characteristics and drug–drug interactions in human epidermal growth factor receptor 2-positive breast cancer treated with trastuzumab deruxtecan: real-world data from the DE-REAL study

**DOI:** 10.1093/oncolo/oyaf402

**Published:** 2026-01-23

**Authors:** Simona Pisegna, Simone Scagnoli, Gabriella Gentile, Antonella Chiavassa, Roberta Caputo, Michelino De Laurentiis, Giuseppe Curigliano, Matteo Lambertini, Francesco Pantano, Armando Orlandi, Antonella Palazzo, Ida Paris, Claudio Vernieri, Beatrice Tedesco, Marianna Giampaglia, Michela Palleschi, Zelmira Ballatore, Daniele Alesini, Giuliana D’Auria, Maria Agnese Fabbri, Luigi Rossi, Giulia Fiscon, Paolo Marchetti, Alessandra Fabi, Andrea Botticelli

**Affiliations:** Department of Experimental Medicine, Sapienza University of Rome, Rome, 00161, Italy; Department of Radiological, Oncological and Pathological Science, Sapienza University of Rome, Rome, 00161, Italy; Department of Radiological, Oncological and Pathological Science, Sapienza University of Rome, Rome, 00161, Italy; Department of Radiological, Oncological and Pathological Science, Sapienza University of Rome, Rome, 00161, Italy; Department of Breast and Thoracic Oncology, Division of Breast Medical Oncology, Istituto Nazionale Tumori IRCCS “Fondazione Pascale, Naples, 80131, Italy; Department of Breast and Thoracic Oncology, Division of Breast Medical Oncology, Istituto Nazionale Tumori IRCCS “Fondazione Pascale, Naples, 80131, Italy; European Institute of Oncology, IRCCS, Milan, 20141, Italy; Department of Oncology and Hemato-Oncology, University of Milan, Milan, 20122, Italy; Department of Internal Medicine and Medical Specialties (DiMI), School of Medicine, University of Genova, Genoa, 16132, Italy; Department of Medical Oncology, U.O.C. Clinica di Oncologia Medica, IRCCS Ospedale Policlinico San Martino, Genoa, 16132, Italy; Medical Oncology, Fondazione Policlinico Universitario Campus Bio-Medico, Rome, 00128, Italy; Department of Medicine and Surgery, Università Campus Bio-Medico di Roma, Rome, 00128, Italy; Comprehensive Cancer Center, Fondazione Policlinico Universitario Agostino Gemelli, IRCCS, Rome, 00168, Italy; Comprehensive Cancer Center, Fondazione Policlinico Universitario Agostino Gemelli, IRCCS, Rome, 00168, Italy; Department of Woman and Child Health and Public Health, Fondazione Policlinico Universitario A. Gemelli IRCCS, Rome, 00168, Italy; Department of Medical Oncology, Fondazione IRCCS Istituto Nazionale Tumori, Milan, 20133, Italy; Department of Oncology and Hematology-Oncology, University of Milan, Milan, 20122, Italy; Medical Oncology Unit, “S. Carlo” Hospital, Potenza, 85100 , Italy; Medical Oncology Unit, “S. Carlo” Hospital, Potenza, 85100 , Italy; Medical Oncology, Breast & GYN Unit, IRCCS Istituto Romagnolo per lo Studio dei Tumori (IRST) “Dino Amadori”, Meldola, 47014, Italy; Medical Oncology Unit, AST Pesaro Urbino, 61121, Italy; UOSD Centro Oncologico S. Spirito e Nuovo Regina Margherita, Ospedale Santo Spirito in Sassia, Rome, 00193, Italy; Department of Medical Oncology, Sandro Pertini Hospital, Rome, 00157, Italy; Department of Oncology and Hematology, Medical Oncology and Breast Unit, Santa Rosa Hospital, Viterbo, 01100, Italy; Multispeciality Department of Oncology, ASL Latina, “Sapienza” University of Rome, Formia, 04011, Italy; Department for the Promotion of Human Science and Quality of Life, San Raffaele Open University, Rome, 00166, Italy; Department of Oncology, Istituto Dermopatico dell’Immacolata IDI-IRCCS, Rome, 00167, Italy; Precision Medicine in Senology Unit, Fondazione Policlinico Universitario A. Gemelli IRCCS, Rome, 00168, Italy; Department of Radiological, Oncological and Pathological Science, Sapienza University of Rome, Rome, 00161, Italy

**Keywords:** trastuzumab deruxtecan, real-world data, HER2-positive breast cancer, body mass index, drug–drug interactions, elderly patients

## Abstract

**Background:**

Trastuzumab deruxtecan (T-DXd) reshaped clinical practice in human epidermal growth factor receptor 2-positive (HER2+) metastatic breast cancer (mBC). The impact of clinical characteristics and drug–drug interactions (DDIs) on outcomes in patients receiving T-DXd is still under investigation.

**Methods:**

We retrospectively analyzed data of patients from the Italian DE-REAL study. Clinical features including age, body mass index (BMI), toxicity and DDIs were assessed and correlated with clinical outcomes. The Drug-PIN software was used to evaluate DDIs.

**Results:**

Among 143 patients, age did not significantly affect progression-free survival (PFS) but influenced overall survival (OS), with younger patients (<65 years) showing better outcomes (median overall survival [mOS]: 12 vs. 10 months, *P* = 0.02). Patients with BMI >25 demonstrated significantly longer PFS (11 vs. 9 months, *P* = 0.04), which was confirmed as independent predictor of better PFS at multivariate analysis (*P* ≤0.05), but experienced higher toxicity rates, particularly nausea (*P* = 0.019). Drug-PIN classification showed no impact on survival outcomes, although patients with high-risk DDIs experienced more nausea and asthenia compared to those with low-risk interactions (*P* = 0.0018 and *P* = 0.003, respectively).

**Conclusion:**

T-DXd efficacy appears consistent across different age groups, although elderly patients showed reduced OS. Higher BMI was associated with improved PFS but increased toxicity. While DDIs did not affect survival outcomes, they influenced specific adverse events. Our results reinforce the efficacy and favorable safety profile of T-DXd in a broad real-world population, including patients with polypharmacy or comorbidities, while highlighting that personalized monitoring and supportive care strategies may be particularly beneficial for elderly patients and those with higher BMI.

Implications for Practice The findings from this real-world analysis provide important clinical guidance for optimizing T-DXd therapy in human epidermal growth factor receptor 2-positive metastatic breast cancer patients. As one of the first studies to comprehensively investigate the role of drug–drug interactions (DDIs) and clinicopathological parameters in predicting response to T-DXd, our results establish a foundation for evidence-based clinical decision-making in this emerging therapeutic landscape. Clinicians should consider implementing enhanced monitoring strategies for elderly patients (≥65 years), potentially through more frequent clinical assessments and proactive supportive care interventions. The identification of body mass index ≥25 kg/m^2^ as an independent predictor of improved progression-free survival, coupled with increased toxicity risk, suggests the need for personalized management approaches including intensified antiemetic protocols and careful symptom monitoring in overweight and obese patients. While DDIs did not significantly impact survival outcomes, the correlation between high-risk DDI classifications and specific adverse events (nausea and asthenia) underscores the value of systematic medication review and DDI assessment tools like Drug-PIN in clinical practice. Overall, these findings support the favorable safety profile of T-DXd, endorsing the maintenance of standard dosing across patient subgroups, while highlighting the importance of individualized supportive care and careful monitoring in particular clinical situations to optimize therapeutic benefit and preserve quality of life in real-world settings.

## Introduction

Current therapies for metastatic breast cancer (mBC) focus on extending survival and effectively managing symptoms.^1^ As known, approximately 15%–20% of primary breast cancers (BC) harbor human epidermal growth factor receptor 2 (HER2) positivity, as defined by an immunohistochemistry result of 3+ or a positive in situ hybridization test.^2^ Historically, HER2+ BC has been associated with an unfavorable prognosis.^3^ Nevertheless, last years have witnessed significant advancements in anti-HER2 therapies, leading to improved outcomes for patients with HER2+ disease.^4^ Despite recent therapeutic improvements, it’s still a challenging reality that most patients will experience disease progression subsequent to anti-HER2 therapy.^5^

Trastuzumab deruxtecan (T-DXd) is a human HER2-targeted antibody-drug conjugate.^6^ T-DXd has shown significant clinical benefits for HER2+ unresectable/metastatic pretreated BC patients in the registrational DESTINY-Breast trials.^7–9^

Initially approved for patients who received at least 2 prior anti-HER-2-based regimens, T-DXd has recently been approved also for pretreated patients with HER2+ BC previously exposed to 1 anti-HER2-based treatment.^10^^,^^11^

According to DB03 results, T-DXd has effectively become the current standard second line after first-line pertuzumab-trastuzumab and taxane.

Ongoing clinical trials are exploring possible use of T-DXd in different setting from early stage to mBC and in combination with other agents, expanding and redesigning its clinical use.^12^

Despite T-DXd’s exceptional and promising role, there are few data on its safety and effectiveness in real world clinical practice.

An age-specific pooled study from DESTINY-Breast01, -02, and -03 showed a T-DXd’s good benefit-risk profile also in patients over 65 years, although with slightly greater toxicity. Nevertheless, optimize and select subgroups with clinical features that mostly can benefit from T-DXd treatment remain challenging.^13^

Clinical characteristics such as age and body mass index (BMI), comorbidities, concomitant medications and frailty, can be critical in clinical outcomes evaluation.^14^

Pharmacokinetics and pharmacodynamics-based drug–drug interactions (DDIs) may change the therapeutic index of anti–cancer treatments, leading to decreased compliance, adverse drug reactions, thereby possibly reducing efficacy.^15–17^

We performed a subgroup analysis of the Italian large retrospective database, the DE-REAL project, focused on clinicopathological characteristics and DDIs in patients treated with T-DXd.^18^

## Methods

### Study population and clinical endpoints

Real-world outcomes of 143 patients with HER2+ mBC treated with T-DXd among 12 Italian referral hospitals were reported in the DE-REAL study. The primary findings of the study demonstrated an objective response rate (ORR) of 68% and a disease control rate (DCR) of 93% in patients with measurable disease, with a real-world PFS of 16 months and median OS of 20 months. Regarding the safety profile, T-DXd appeared well tolerated with adverse events (AEs) of any grade reported in 59% of patients, as previously reported.^18^ In this context, the present analysis aims to further investigate demographic and clinicopathological data among patients receiving T-DXd, including age, menopausal status, BMI, site of metastases, therapy line, type and toxicity grading, as well as medication interactions. Subsequently, this study evaluates the association between these clinical variables and survival outcomes, specifically real-world progression-free survival (rwPFS) and overall survival (OS).

### Patient’s stratification

The study cohort was stratified according to age utilizing predetermined thresholds: patients younger than 65 years versus those 65 years of age or older, and concurrently, subjects <75 years versus those 75 years or above.^19^ BMI status was calculated using the weight/height2 formula (kg/m^2^). BMI values *≥*25 kg/m^2^ value were used as cutoff to identify patients with overweight/obesity.^20^ Regarding concomitant medications, patients were categorized into 3 groups based on the number of concurrent medications at baseline: no concomitant medications (0 medications), low polypharmacy (≤3 medications), and high polypharmacy (>3 medications).Visceral disease was defined by the presence of at least one metastatic lesion within hepatic, pulmonary, cerebral, and/or peritoneal tissues. Conversely, patients presenting exclusively with bone, lymphatic, and/or soft tissue metastases were categorized as having non–visceral disease.

Treatment-related AEs collected in the referral centers were categorized and graded according to the National Cancer Institute Common Terminology Criteria for Adverse Events (NCI CTCAE), version 5.0.19.^21^

Radiological assessments were carried out every 3–4 months, without a fixed schedule, as per routine clinical practice. Real-world PFS (rwPFS) was defined as the time from the first T-DXd administration to the time of the radiological progression or death. OS was defined as the time from the first treatment to the death.

Drug-PIN (Personalized Interactions Network) was utilized to evaluate the pharmacological interactions of medications. Drug-PIN is a medical software that analyzes clinical, biochemical, and demographic data from patients and combines it with a simultaneous DDIs profile, allowing researchers to investigate the effects of active and/or pro-drug forms and identify patients at high risk of toxicity with oncological therapies.^22^

Furthermore, the software collects and analyzes clinical data (age, weight, liver, and renal function), DDIs, and, if available, patients’ genomic profiles.

The Drug-PIN system produces a numerical score (Drug-PIN score) that indicates the risk of DDIs, as well as a tier (Drug-PIN light: green, yellow, dark yellow, and red), which indicates the risk of interactions and whether the concomitant drugs need to be changed with a drug reconciliation process. The output ranges are separated as follows: Drug-PIN scores range from 0 to 20 (green light) indicating no interactions; 20-30 (yellow light) indicating low-risk DDIs; 30-70 (dark yellow light) indicating intermediate-high risk DDIs; and ≥70 (red light) indicating high-risk interactions. The software used machine learning techniques to perform a multi-pass analysis, integrating data for each new element added to the patient’s record. DRUG-Pin score and tier were calculated for each patient with and without T-DXd addition.

The drug interaction test was carried out using the concomitant medications provided at baseline. For Drug-PIN analysis, patients were stratified into 2 categories based on DDI risk assessment: no/low-risk DDIs (green Drug-PIN light) versus any-risk DDIs (yellow/dark yellow/red Drug-PIN light combined).

### Statistical analysis

Median and range with number (percentages) or mean ± standard deviations were used for summarizing data. Continuous variables were analyzed using independent *T* Student’s test, while categorical variables were studied using chi-square or Fisher’s exact test. The prognosis of each group of patients was examined by Kaplan–Meier survival estimators, and the survival outcomes of the 2 groups were compared by log-rank tests. Log rank *P*-values less than or equal to 0.05 were considered as statistically significant. Univariate and multivariate cox proportional hazards models were also used to assess the association between clinical, biological, pathological variables, and survival outcomes.

### Ethical approval

The study was approved by the Institutional Review Board (IRB) of the coordinating center Sapienza–Policlinico Umberto I of Rome (examination number: 0181/2022) and all other institutions. All procedures were performed in accordance with the principles of the Declaration of Helsinki and with institutional and national standards on human experimentation. A specific informed consent form was signed by all patients alive at the time of the study approval.

## Results

### Study population

The De-REAL study enrolled 143 patients treated with T-DXd between April 2021 and October 2022, with a median age of 66 years (range: 37–90 years) and a median age at T-DXd start of 57 years. Of these, 4 patients (3%) were male, while 139 patients (97%) were female, with 64 patients in postmenopausal status. Among the study population, 108 patients (75%) presented with estrogen receptor (ER) positive disease and 35 patients (25%) with ER negative mBC. Regarding age distribution, 106 patients were aged <65 years and 37 had ≥ 65 years. Overall, 13 patients (9%) were aged ≥75 years. BMI analysis revealed that 55 patients (38%) had a BMI *≥*25, whereas 88 patients (62%) had a BMI <25 kg/m^2^. T-DXd was administered as first or second-line in 20 patients (14%), while 123 patients (86%) received treatment as third or subsequent lines. Eighty-four patients had visceral disease, while 59 showed no visceral involvement, respectively. As previously reported, AEs were documented in 59% of subjects (*n* = 84). Gastrointestinal toxicity, primarily manifesting as nausea, constituted the predominant AE, with an incidence of 33% across all severity grades (*n* = 47). Neutropenia and fatigue were also frequent, occurring both in 21% of cases (*N* = 30, any grade). Mild to moderate AEs (grades 1-2) were documented in approximately two-thirds of the cohort (69%, *n* = 58). Dose reductions were implemented in 26% of participants (*n* = 37), whereas permanent discontinuation of T-DXd therapy was required only in 3% of cases. Regarding comorbidities, 105 patients (73%) reported no significant comorbidities, while 38 patients (27%) had at least one comorbidity. Concerning concomitant medications, 81 patients (57%) were not taking any concomitant medications, 50 patients (35%) were taking ≤3 medications, and 12 patients (8%) were taking >3 concomitant medications respectively. Median Drug-PIN score was 6.0 (range 0-186). Of 142 evaluable patients, 126, 11, 3, and 2 were in the green, yellow, dark yellow, and red tier respectively, with most of patients falling in the no/low-risk DDI group. No significant modifications in Drug-PIN score and tier were noted when adding T-DXd to patients’ concomitant therapy (8.5 vs. 6.0, *P* = 0.47). Patients’ characteristics are reported in Table 1.

**Table 1.** Patient characteristics.

Characteristic	*N* (%)
**Age (years)**	
<65	106 (74)
≥65	37 (26)
<75	130 (91)
≥75	13 (9)
**Menopausal status**	
Premenopausal	75 (54)^a^
Postmenopausal	64 (46)^a^
**ER status**	
Positive	108 (75)
Negative	35 (25)
**Body mass index (BMI)**	
<25 kg/m²	88 (62)
≥25 kg/m²	55 (38)
**Visceral disease**	
Yes	84 (59)
No	59 (41)
**T-DXd treatment line**	
First or second line	20 (14)
≥3rd line	123 (86)
**Adverse events (AEs)**	
Any AE	84 (59)
Nausea (any grade)	47 (33)
Neutropenia (any grade)	30 (21)
Fatigue (any grade)	30 (21)
Dose reduction	37 (26)
Permanent discontinuation	4 (3)
**Toxicity grade^b^**	
G1/G2	58 (69)
G3/G4	26 (31)
**Drug-PIN score^c^**	
Median (range)	6.0 (0-186)
**Drug-PIN light^c^**	
Green	126 (89)
Yellow	11 (8)
Dark yellow	3 (2)
Red	2 (1)
**Concomitant medications**	
No	81 (57)
≤3	50 (35)
>3	12 (8)
**Comorbidities**	
No	105 (73)
Yes	38 (27)

a Percentage calculated among female patients only.

b Percentage calculated among patients with any adverse event (*N* = 84).

c Percentage calculated among evaluable patients (*N* = 142).

Abbreviations: AE, adverse event; ER, estrogen receptor; *N*, number of patients; NA, not available; T-DXd, trastuzumab deruxtecan.

### Outcomes

**Figure 1.**
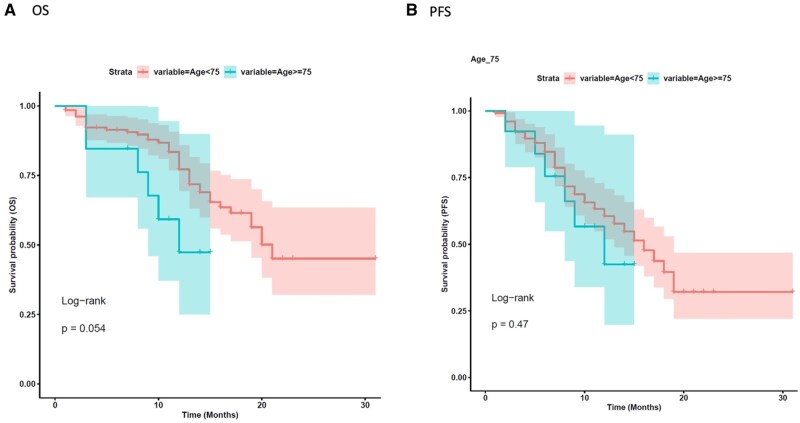
Kaplan–Meier analysis for age stratification <75 vs. ≥75 years. The correlation between variable value and patient survival was examined as overall survival (OS) (A) and progression-free survival (PFS) (B).

**Figure 2.**
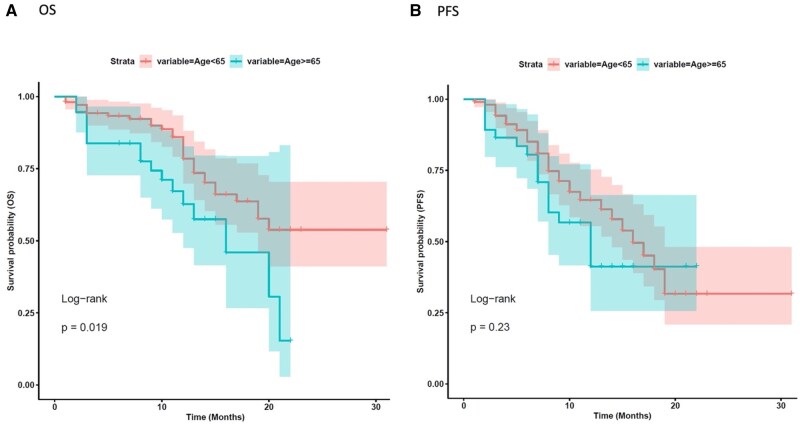
Kaplan–Meier analysis for age stratification <65 vs. ≥65 years. The correlation between variable value and patient survival was examined as overall survival (OS) (A) and progression-free survival (PFS) (B).

**Figure 3.**
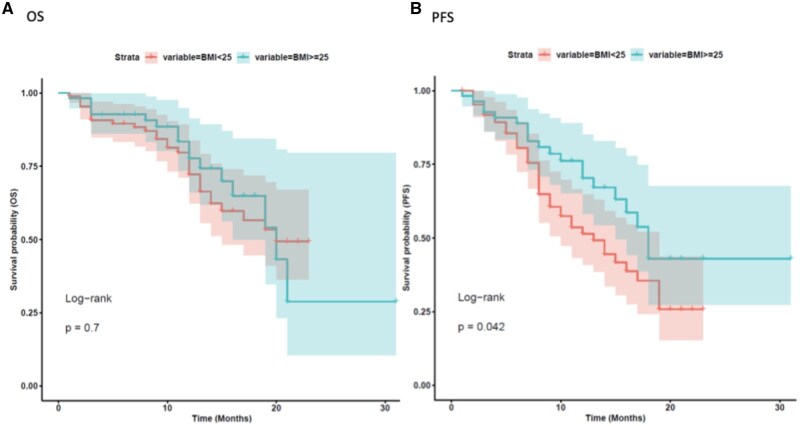
Kaplan–Meier analysis for body mass index (BMI) stratification ≤25 vs. >25. The correlation between variable value and patient survival was examined as overall survival (OS) (A) and progression-free survival (PFS) (B).

We initially evaluated the influence of key clinical features on median real-world progression free survival (mrwPFS) and median overall survival (mOS). With a median follow up of 12 months, age did not significantly affect rwPFS in our analysis, both in the age-based subgroups <75 versus ≥75 years, and in the subgroup <65 versus ≥65 (mrwPFS 10 vs. 9 months, HR 1.37 [0.59-3.2], *P* = 0.47 and 10 vs. 8 months, HR 1.41 [0.81-2.5], *P* = 0.22, respectively, Figures 1B and 2B). However, patients under 75 years a non–significant trend toward improved OS compared to those aged ≥75 (mOS 12 vs. 10 months, HR 2.32 [0.97-5.6], *P* = 0.054 (Figure 1A). Similarly, patients younger than 65 years had a more favorable mOS compared to those aged 65 years or older (mOS 12 vs. 10 months; HR 2.06 [1.1-3.8], *P* = 0.02, Figure 2A). Comparative analysis of baseline characteristics across age groups revealed that most variables showed no significant associations, with the exception of expected age-related differences in clinical practice. Predictably, elderly patients demonstrated significantly higher rates of comorbidities and polypharmacy. Specifically, patients aged ≥65 years had significantly higher rates of comorbidities compared to younger patients (48.6% vs. 18.9%, *P* = 0.001), with similar findings observed in the ≥75 years subgroup (54% vs. 23.85%, *P* = 0.045) (Tables S1 and S^2^). BMI significantly affected rwPFS in the overall population. Patients with BMI *≥*25 kg/m^2^ exhibited longer mrwPFS compared to those with BMI <25 kg/m^2^ (11 vs. 9 months, HR = 0.58 [0.34-0.98], *P* = 0.04). However, BMI did not significantly influence OS (mOS 12 vs. 11 months, HR 0.88 [0.48-1.6], *P* = 0.7, Figure 3).

**Figure 4.**
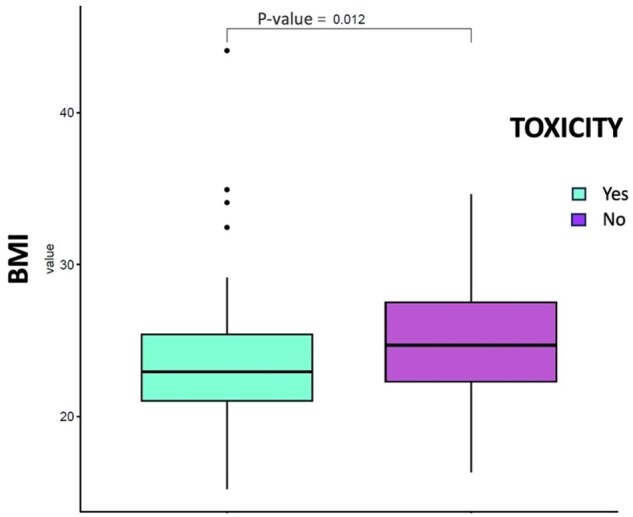
Boxplot of the body mass index (BMI) variable in patients without toxicity and in patients with toxicity by performing a Student *t*-test. Patients with toxicity have higher value of BMI (*P* = 0.012).

**Figure 5.**
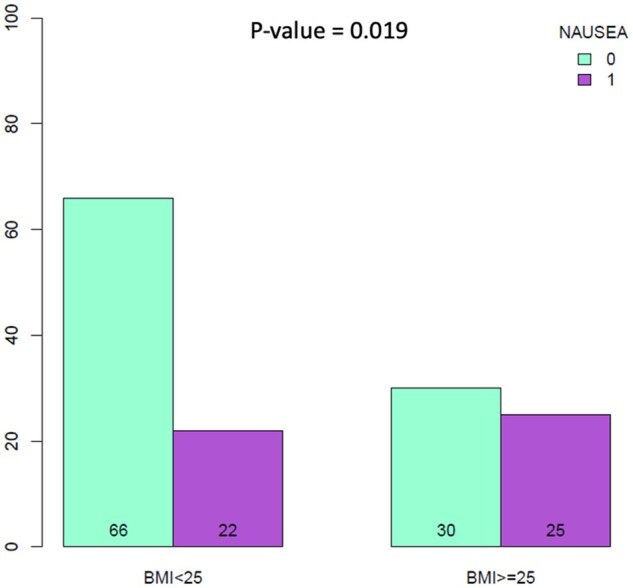
Barplot illustrating the contingency table obtained for the body mass index (BMI) variable, classified as patients with BMI < 25 and BMI ≥ 25 with respect to the patients with nausea and without nausea by performing a chi-square test. The two variables (body mass index [BMI] and nausea) are related to each other (*P* = 0.019).

Notably, patients experiencing toxicities had a significantly higher median BMI than those without toxicities (24.69 vs. 22.95; *P* = 0.01, Figure 4). Among all treatment-related AEs analyzed, nausea was the only toxicity that demonstrated a significant association with BMI status. Specifically, patients with BMI ≥25 kg/m^2^ experienced significantly higher rates of nausea compared to those with BMI <25 kg/m^2^ (*P* = 0.019, Figure 5). However, when comparing patients with BMI *≥*25 versus <25 kg/m^2^ across different severity grades, no significant differences were observed between Grade 1-2 versus Grade 3-4 nausea (*P* = 1.0). No significant association were found between BMI status and other treatment-related toxicities, including hematological toxicity, interstitial lung disease (ILD), or hepatotoxicity (*P* > 0.05).

**Figure 6.**
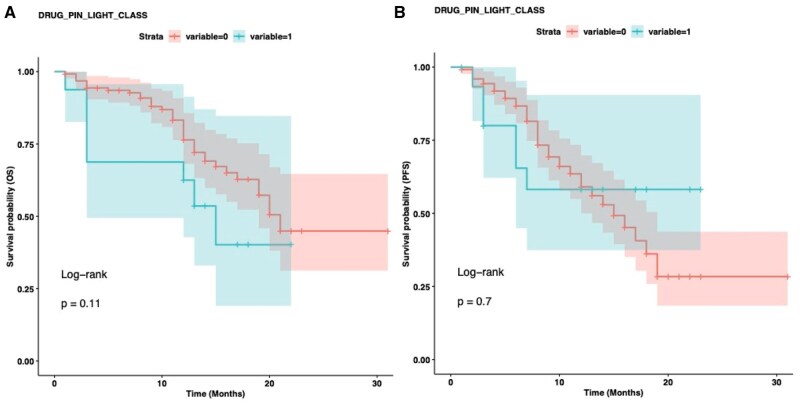
Kaplan–Meier analysis for Drug-PIN class (green vs. yellow/dark yellow/red light). Variable 0 represents green tier (no/low-risk drug–drug interactions [DDIs]) and variable 1 represents combined yellow/dark yellow/red tiers (any-risk DDIs). The correlation between Drug-PIN classification and patient survival was examined as overall survival (OS) (A) and progression-free survival (PFS) (B).

Conversely, other clinical features such as menopausal status, the number of prior therapy lines, toxicity (yes vs. no) and toxicity grade (G1/G2 vs. G3/G4), visceral disease, ER status, dose reduction and Drug-PIN light did not significantly impact both on rwPFS and OS (all *P*-value > 0.05, Table S4). At multivariate analysis, only BMI *≥*25 remained significantly associated with longer rwPFS (*P* ≤ 0.05; Figure S1, see online supplementary material for a color version of this figure). We also explored the role of concomitant medications and potential DDIs. Patients with higher Drug-PIN risk classifications (yellow/dark yellow/red) were significantly more likely to have multiple concomitant medications (*P* < 0.001) and associated comorbidities (*P* < 0.001) compared to those in the green category (Table S3). Baseline Drug-PIN classifications (green light vs. yellow/dark yellow/red light) showed no significant impact on OS (mOS 11 vs. 12 months, HR: 1.87 [0.87-4], *P* = 0.11) or rwPFS (mrwPFS 10 vs. 9.5 months, HR: 0.84 [0.36-2], *P* = 0.69, Figure 6). No significant association was detected within Drug-PIN light and occurrence of any toxicity (*P* = 0.2) and toxicity grade (G1/G2 vs. G3/G4, *P* = 0.3). However, some specific AEs such as nausea and asthenia were significantly more frequent among patients with any-risk DDIs (ie, yellow, orange, or red categories) compared to those with no-risk DDIs (*P* = 0.018 and *P* = 0.003, respectively; Figure S2, see online supplementary material for a color version of this figure).

## Discussion

The De-REAL study provides valuable insights into the impact of clinical and pharmacological factors on the outcomes of patients treated with T-DXd. Interestingly, rwPFS remained relatively consistent across the age spectrum, with no statistically significant differences observed between patients categorized as <75 ≥75 years (mrwPFS 10 vs. 9 months, *P* = 0.47) or <65 versus ≥65 years (mrwPFS 10 vs. 8 months, *P* = 0.22). This observation suggests that the tumor-controlling efficacy of T-DXd is largely preserved regardless of patient age. In contrast, our findings indicate that age significantly influenced OS: a trend toward worse OS was observed in patients aged ≥75 and ≥65 years compared to their younger counterparts (mOS 12 vs. 10 months, *P* = 0.054 and mOS 12 vs. 10 months, *P* = 0.02).

This finding aligns with prior studies suggesting that elderly patients often present with more comorbidities and reduced physiological reserves, potentially limiting their ability to receive subsequent therapies upon disease progression.^23^^,^^24^ Data from ESME database confirms that only 65% of women aged 70+ with HER2+ mBC receive a standard first-line treatment due to comorbidities and frailty with lower PFS and OS compared to the patients <70 years.^25^ A specific age pooled analysis examined 44 (23.9%), 85 (20.9%), and 49 (18.8%) patients aged ≥65 who received T-DXd in DESTINY-Breast01, DESTINY-Breast02, and DESTINY-Breast03. The results of this pooled analysis also show that T-DXd has a positive benefit-risk profile in patients aged 65 and older, although with higher toxicity.^13^ However, older patients remain underrepresented in most of randomized clinical trials, creating a significant knowledge gap in understanding drug efficacy, optimal dosing strategies, and management of treatment-related toxicities in geriatric oncology.^26^ While T-DXd appears to provide initial benefit across all age groups in our analysis, the reduced OS in elderly patients, compared to youngers, suggests the need for careful monitoring during therapy, and potentially more proactive supportive care measures. Additionally, the consideration of age-adapted dosing strategies may be warranted, particularly in patients ≥75 years, to optimize the risk-benefit ratio. It is essential to emphasize that the observed differences in OS between age groups do not reflect inherent differences in T-DXd drug efficacy, but rather real-world complexities and patient-specific vulnerabilities. Particularly, patients aged ≥75 years may present with reduced physiological reserves and increased comorbidities that affect survival outcomes independently of treatment efficacy. The consistent efficacy of T-DXd in disease progression control (rwPFS) across all age groups supports this interpretation, suggesting that the drug’s antitumor activity remains preserved regardless of patient age.

Interestingly, BMI emerged as a significant factor influencing rwPFS, with patients having a BMI ≥25 kg/m^2^ demonstrating longer rwPFS compared to those with a BMI <25 (mrwPFS 11 vs. 9 months, *P* = 0.04). This finding suggests that increased body mass may confer a therapeutic advantage with respect to immediate disease control during T-DXd therapy. However, this apparent benefit did not translate into OS improvements, as no significant difference in OS was observed between the BMI categories (mOS 12 vs. 11 months, *P* = 0.7). Additionally, although obesity is traditionally thought to be a pro-carcinogenic state and increase the risk of BC, in some situations, being overweight or obese appears to be related to better clinical outcomes, possibly reflecting the negative prognostic role of malnourishment and cachexia in this setting.^27^ These findings align with a large-scale pooled analysis of four pivotal phase III trials (CLEOPATRA, MARIANNE, EMILIA, and THERESA) encompassing 3496 HER2+ mBC patients, which demonstrated superior PFS and OS outcomes in overweight and obese patients compared to those with normal BMI.^28–30^ Intriguingly, multivariate analysis confirmed the protective effect of BMI *≥*25 on rwPFS (*P* ≤ 0.05), even after adjusting for potential confounding factors. Although pharmacokinetic analysis suggest that body weight seem not to have a clinically meaningful effect on T-DXd distribution or released deruxetcan (DXd), the increased volume of distribution in patients with higher BMI could potentially lead to more sustained therapeutic levels of the drug, while simultaneously resulting in a higher incidence of manageable AEs. Additional research is warranted to gain a deeper understanding of how BMI, body composition, and fat mass influence the efficacy of T-DXd and antibody-drug conjugates (ADCs) more broadly.^31^

Notably, our analysis demonstrated that patients with higher BMI experienced significantly more treatment-related toxicities. The median BMI was markedly higher in patients who developed AEs compared to those who did not (24.69 vs. 22.95, *P* = 0.01). Specifically, patients with BMI *≥*25 kg/m^2^ exhibited an increased incidence of nausea, which represents one of the most common toxicities associated with T-DXd therapy, suggesting a complex relationship between BMI and clinical outcomes in patients treated with T-DXd as hypothesized above. Notably, this association was not dependent on toxicity grade (*P* = 1). This finding suggests that higher BMI primarily influences the likelihood of developing nausea as an AE rather than determining its severity. This observation adds nuance to our understanding of the BMI-toxicity relationship in T-DXd therapy. The consistent toxicity regarding severity grades supports the hypothesis that higher BMI affects drug distribution and initial toxicity susceptibility without fundamentally altering the dose-response relationship for severe AEs. At the same time, obesity-related inflammation likely sensitizes gastrointestinal neural pathways to drug effects and the bystander effect of T-DXd may be amplified in adipose tissue, increasing systemic toxicity. Proactive antiemetic protocols might be especially important for patients with elevated BMI receiving T-DXd. Moreover, the observed toxicity profile raises the question of whether BMI-adjusted dosing strategies might optimize the therapeutic index of T-DXd in certain patient populations. In contrast, other clinical parameters, including menopausal status, number of prior therapy lines, and dose reduction, did not demonstrate significant associations with survival outcomes in our cohort. This suggests that T-DXd’s efficacy remains consistent across these clinical subgroups, supporting its robust therapeutic potential in diverse patient populations, even in heavily pretreated patients.

These findings contribute to the growing body of evidence regarding the impact of body composition on ADC efficacy and tolerability, while highlighting the importance of careful monitoring and supportive care in patients with higher BMI to optimize the risk-benefit ratio of T-DXd therapy.

The impact of concomitant medications and DDIs on clinical outcomes was also explored. Pharmacokinetics and pharmacodynamics-based DDIs in cancer patients using many concomitant medications may modify the therapeutic index of anti–cancer treatments, resulting in poor compliance, undesirable adverse drug reactions and treatment failure.^16^

In our study, the Drug-PIN classification at baseline showed no apparent impact on survival outcomes. However, patients in the green light Drug-PIN light category were less likely to experience nausea or asthenia compared to those with higher interaction risk levels. Some methodological considerations should be acknowledged regarding our DDI analysis. The decision to combine yellow, dark yellow, and red Drug-PIN categories into a single “any-risk” group was necessitated by small sample sizes in individual higher-risk strata. While this approach enabled statistical analysis, it may potentially mask differences between intermediate and high-risk interaction profiles, representing a limitation that should be considered when interpreting these results. The value of evaluating DDIs through dedicated software platforms in real-world BC populations has been recently highlighted by other studies.

While the current analysis was not specifically intended to validate the Drug-PIN platform, its application is grounded in prior oncologic research. Multiple studies in breast, colorectal and melanoma cancers have confirmed its accuracy and reproducibility in detecting clinically meaningful DDIs, supporting its integration in this real-world BC population.^32–35^

Though our study showed no significant impact of baseline Drug-PIN classifications on survival outcomes, patients with higher Drug-PIN scores experienced worse clinical outcomes, particularly in terms of PFS in the AB-ITALY study. This difference might be explained by the distinct pharmacological properties and metabolic pathways of T-DXd compared to abemaciclib, as well as the different concomitant medications typically used in these treatment settings. The high molecular weight of the antibody component and the lipophilic nature of T-DXd payload may result in different affect exposure patterns.^30^ This characteristic might potentially buffer the impact of DDIs on systemic exposure in overweight patients treated with T-DXd compared to other anti-cancer agents which show more direct pharmacokinetic interactions with concomitant medications.

However, both studies underscore the importance of systematic DDI assessment in clinical practice, as evidenced by the correlation between Drug-PIN classification and specific toxicity patterns. These findings collectively suggest that while the clinical impact of DDIs may vary across different therapeutic agents, tools like Drug-PIN can provide valuable insights for toxicity management and treatment optimization in real-world populations, in order to improve tolerability and patient quality of life.

Moreover, the specific pharmacological properties of T-DXd as an antibody-drug conjugate may inherently lead to fewer direct DDIs compared to small-molecule therapies, contributing to the low prevalence of high-risk DDIs observed in our cohort. This finding supports the notion that T-DXd exhibits a favorable safety profile in real-world clinical practice, with minimal pharmacological interference even among patients receiving concomitant medications. Although our analysis was not specifically designed to identify individual drugs requiring caution and did not reveal any signal for particular medications, the overall safety signal reinforces the clinical manageability of T-DXd and its suitability for use across heterogeneous, real-world populations.

Some limitations should be acknowledged when interpreting our findings. First, the retrospective nature of this analysis inherently introduces potential selection and recall bias. Additionally, the real-world setting might have resulted in missing data and heterogeneous assessment schedules across participating centers. The relatively small sample size in certain subgroups, particularly older patients aged ≥75 years and those with BMI <25 kg/m^2^, may have limited our ability to detect statistically significant differences in outcomes. Moreover, BMI represents a measure of body composition that does not distinguish between adipose and lean tissue distribution and longitudinal BMI measurements were not consistently available, precluding analysis of how weight changes during treatment might influence outcomes. Furthermore, the assessment of DDIs was based on baseline medications only, and changes in concomitant treatments during T-DXd therapy were not captured in our analysis. The relatively small number of patients with any DDI risk (*n* = 16, 11% of the total cohort) represents an additional limitation that constrains the generalizability of our DDI-related findings. This limited sample size reflects the specific pharmacological characteristics of T-DXd, which may have fewer conventional DDIs due to its antibody-drug conjugate structure. Collectively, our results reinforce the efficacy and favorable safety profile of T-DXd in a broad real-world population, underscoring its clinical manageability even in patients with polypharmacy or comorbidities. Although no specific clinical scenarios or concomitant drugs requiring particular caution were identified, our findings suggest that certain patient-related factors, such as higher BMI or advanced age, may influence treatment tolerability and should be considered when tailoring T-DXd therapy in daily practice. Future studies should aim to further characterize the relationships between clinical features, toxicity profiles, and the role of longitudinal DDI assessment to better guide clinical decision-making and improve patients’ outcomes. Larger cohorts specifically enriched for patients with high-risk DDI profiles will be necessary to validate these preliminary findings and better characterize the clinical impact of drug interactions in T-DXd therapy. Additionally, prospective studies focusing exclusively on patients with established high-risk DDIs could provide more definitive insights into the clinical significance of drug interactions in this therapeutic context. Moreover, further research focused on prospectively validating the impact of BMI on T-DXd efficacy and toxicity could potentially lead to optimized dosing strategies for specific patient subgroups.

## Supplementary Material

oyaf402_Supplementary_Data

## Data Availability

The data that support the findings of this study are available from the corresponding author upon reasonable request.
