# Supplementary material for: Clinical characteristics and drug–drug interactions in human epidermal growth factor receptor 2-positive breast cancer treated with trastuzumab deruxtecan: real-world data from the DE-REAL study
Source: Oncologist. 2026 Jan 23;31(2):oyaf402. doi: 10.1093/oncolo/oyaf402 (PMC12848230; doi:10.1093/oncolo/oyaf402)
Supplement: oyaf402_Supplementary_Data [file oyaf402_supplementary_data.zip › Supp. Table 3.docx]

**Suppl. Table 3.** Patient Characteristics according to drug-pin light.

| **Characteristic** | **Drug Pin Green**  **N (%)**  **126 (89)** | **Drug Pin**  **Yellow- Dark Yellow- Red**  **N (%)**  **16 (11)** | **P-value** |
| --- | --- | --- | --- |
| **ER status** |  |  | 0.734 |
| Positive | 93 (73.81) | 13 (81.25) |  |
| Negative | 33 (26.19) | 3 (18.75) |  |
| **Body Mass Index (BMI)** |  |  | 0.478 |
| < 25 kg/m² | 79 (63) | 8 (50) |  |
| ≥ 25 kg/m² | 47 (37) | 8 (50) |  |
| **Visceral disease** |  |  | 0.602 |
| Yes | 76 (60.32) | 8 (50) |  |
| No | 50 (39.68) | 8 (50) |  |
| **T-DXd treatment line** |  |  | 0.565 |
| 1st or 2nd line | 19 (15.08) | 1 (6.25) |  |
| ≥ 3rd line | 107 (84.92) | 15 (93.75) |  |
| **Adverse events (AEs)** |  |  |  |
| Any AE | 70 (55.56) | 12 (75) | 0.225 |
| Nausea (any grade) | 37 (29.37) | 10 (62.5) | 0.012 |
| Neutropenia (any grade) | 26 (20.63) | 4 (25) | 0.938 |
| Fatigue (any grade) | 22 (17.46) | 7 (43.75) | 0.033 |
| Dose reduction | 32 (25.40) | 5 (31.25) | 0.841 |
| Permanent discontinuation | 61 (48.41) | 6 (37.5) | 1 |
| **Toxicity grade**** |  |  | 0.281 |
| G1/G2 | 105 (83.33) | 11 (68.75) |  |
| G3/G4 | 21 (16.67) | 5 (31.25) |  |
| **Concomitant medications** |  |  | 3.74*10^−06^ |
| No | 81 (64.29) | 0 |  |
| ≤3 | 40 (31.75) | 10 (62.5) |  |
| >3 | 5 (3.97) | 6 (37.5) |  |
| **Comorbidities** |  |  | 1.51*10^−05^ |
| No | 100 (79.37) | 4 (25) |  |
| Yes | 26 (20.63) | 12 (75) |  |

P refers to p-values obtained from ChiSquare or Fischer’s Exact test. **Percentage calculated among patients with any adverse event (N=84)
