# Supplementary material for: Clinical characteristics and drug–drug interactions in human epidermal growth factor receptor 2-positive breast cancer treated with trastuzumab deruxtecan: real-world data from the DE-REAL study
Source: Oncologist. 2026 Jan 23;31(2):oyaf402. doi: 10.1093/oncolo/oyaf402 (PMC12848230; doi:10.1093/oncolo/oyaf402)
Supplement: oyaf402_Supplementary_Data [file oyaf402_supplementary_data.zip › Supp. Table 4.docx]

**Suppl.Table 4** Survival Outcomes Based on Dose Modifications, Toxicity, and Drug Interactions

|  | **mPFS** | **HR (CI 95%)** | **p-value** | **mOS** | **HR (CI 95%)** | **p-value** |
| --- | --- | --- | --- | --- | --- | --- |
| **Dose reduction** | 9 | 0.885 (0.51-1.5) | 0.663287245 | 11 | 0.62 (0.31-1.3) | 0.184868 |
| **Full Dose** | 10 |  |  | 14 |  |  |
| **Green Drug Pin Light** | 10 | 0.846 (0.36-2) | 0.696602994 | 11 | 1.87 (0.87-4) | 0.111361 |
| **Orange or Yellow or Red Drug Pin Light** | 9.5 |  |  | 12 |  |  |
| **Toxicity** | 9 | 0.806 (0.49-1.3) | 0.40424603 | 10 | 0.913 (0.5-1.7) | 0.768499 |
| **No Toxicity** | 10 |  |  | 13 |  |  |
| **Toxicity Grade 1 and Grade 2** | 10 | 1.21 (0.67-2.2) | 0.521557851 | 11 | 1.13 (0.56-2.3) | 0.727284 |
| **Toxicity Grade 3 and Grade 4** | 8.5 |  |  | 12 |  |  |
