# Supplementary material for: Clinical characteristics and drug–drug interactions in human epidermal growth factor receptor 2-positive breast cancer treated with trastuzumab deruxtecan: real-world data from the DE-REAL study
Source: Oncologist. 2026 Jan 23;31(2):oyaf402. doi: 10.1093/oncolo/oyaf402 (PMC12848230; doi:10.1093/oncolo/oyaf402)
Supplement: oyaf402_Supplementary_Data [file oyaf402_supplementary_data.zip › Supp.Table 1.docx]

| **Characteristic** | **<65**  **N (%)**  **106 (74)** | **≥65**  **N (%)**  **37 (26)** | **P-value** |
| --- | --- | --- | --- |
| **ER status** |  |  | 0.401 |
| Positive | 81 (76.4) | 25 (67.6) |  |
| Negative | 25 (23.6) | 12 (32.4) |  |
| **Body Mass Index (BMI)** |  |  | 0.094 |
| < 25 kg/m² | 70 (66) | 18 (49) |  |
| ≥25 kg/m² | 36(34) | 19 (51) |  |
| **Visceral disease** |  |  | 0.01 |
| Yes | 70 (66) | 22 (59.5) |  |
| No | 36 (34) | 15 (40.5) |  |
| **T-DXd treatment line** |  |  | 0.86 |
| 1st or 2nd line | 14 (13.2) | 6 (16.2) |  |
| ≥ 3rd line | 92 (86.8) | 31 (83.8) |  |
| **Adverse events (AEs)** |  |  |  |
| Any AE | 62 (58.5) | 21 (56.8) | 1 |
| Nausea (any grade) | 35 (33) | 12 (32.4) | 1 |
| Neutropenia (any grade) | 21 (19.8) | 9 (24.3) | 0.73 |
| Fatigue (any grade) | 25 (23.6) | 5 (13.5) | 0.29 |
| Dose reduction | 27 (25.5) | 9 (24.3) | 1 |
| Permanent discontinuation | 51 (48.1) | 17 (46) | 1 |
| **Toxicity grade** |  |  | 0.38 |
| G1/G2 | 89 (84) | 28 (75.7) |  |
| G3/G4 | 17 (16) | 9 (24.3) |  |
| **Drug-PIN score°** |  |  |  |
| Median | 3 (1.7-190.1) | 3 (1-78.9) | 0.51 |
| **Drug-PIN light°** |  |  | 0.37 |
| Green | 96 (91.5) | 30 (81.1) |  |
| Yellow | 6 (5.7) | 5 (13.5) |  |
| Dark yellow | 2 (1.9) | 1 (2.7) |  |
| Red | 1 (0.9) | 1 (2.7) |  |
| **Concomitant medications** |  |  | 0.34 |
| No | 63 (59.4) | 18 (48.6) |  |
| ≤3 | 36 (34) | 14 (37.8) |  |
| >3 | 7 (6.6) | 5 (13.5) |  |
| **Comorbidities** |  |  | 0.001 |
| No | 86 (81.1) | 19 (51.4) |  |
| Yes | 20 (18.9) | 18 (48.6) |  |

**Suppl. Table 1.** Patient Characteristics according to age-groups (<65 vs ≥65).

Legend N: number of patients; ER: estrogen receptor; T-DXd: trastuzumab deruxtecan; AE: adverse event; NA: not available. P refers to p-values obtained from ChiSquare or Fischer’s Exact test for categorical variables, and from Student’s t-test for continuous variable (i.e., drug PIN score).
