# Supplementary material for: Clinical characteristics and drug–drug interactions in human epidermal growth factor receptor 2-positive breast cancer treated with trastuzumab deruxtecan: real-world data from the DE-REAL study
Source: Oncologist. 2026 Jan 23;31(2):oyaf402. doi: 10.1093/oncolo/oyaf402 (PMC12848230; doi:10.1093/oncolo/oyaf402)
Supplement: oyaf402_Supplementary_Data [file oyaf402_supplementary_data.zip › Suppl. Fig. 1 dereal.docx]

**Suppl. Fig.1**. Forest plot of multivariate Cox regression analysis for progression-free survival (PFS). Hazard ratios (HR) with 95% confidence intervals are shown for each variable. BMI >25 was significantly associated with improved PFS (HR <1, p<0.05).
