# Supplementary material for: Clinical characteristics and drug–drug interactions in human epidermal growth factor receptor 2-positive breast cancer treated with trastuzumab deruxtecan: real-world data from the DE-REAL study
Source: Oncologist. 2026 Jan 23;31(2):oyaf402. doi: 10.1093/oncolo/oyaf402 (PMC12848230; doi:10.1093/oncolo/oyaf402)
Supplement: oyaf402_Supplementary_Data [file oyaf402_supplementary_data.zip › Suppl.Fig. 2 dereal.docx]

**Suppl.Fig.2** Barplot illustrating the contingency table obtained for Drug pin light variable, classified as patients with green light and patients with yellow, orange or red light with respect to the patients with and without nausea [A] and asthenia [B].
