# Supplementary material for: Clinical characteristics and drug–drug interactions in human epidermal growth factor receptor 2-positive breast cancer treated with trastuzumab deruxtecan: real-world data from the DE-REAL study
Source: Oncologist. 2026 Jan 23;31(2):oyaf402. doi: 10.1093/oncolo/oyaf402 (PMC12848230; doi:10.1093/oncolo/oyaf402)
Supplement: oyaf402_Supplementary_Data [file oyaf402_supplementary_data.zip › Suppl.Table 2 .docx]

**Suppl. Table 2.** Patient Characteristics according to age-groups (<75 vs ≥75).

| **Characteristic** | **<75**  **N (%)**  **130 (91)** | **≥75**  **N (%)**  **13 (9)** | **P** |
| --- | --- | --- | --- |
| **ER status** |  |  | 0.93 |
| Positive | 97 (74.6) | 9 (69.23) |  |
| Negative | 33 (25.4) | 4 (30.77) |  |
| **Body Mass Index (BMI)** |  |  | 0.37 |
| < 25 kg/m² | 82 (63) | 6 (46) |  |
| ≥ 25 kg/m² | 48 (37) | 7 (54) |  |
| **Visceral disease** |  |  | 0.187 |
| Yes | 80 (61.5) | 5 (38.5) |  |
| No | 50 (38.5) | 8 (61.5) |  |
| **T-DXd treatment line** |  |  | 1 |
| 1st or 2nd line | 18 (13.8) | 2 (15.38) |  |
| ≥ 3rd line | 112 (86.2) | 11 (84.62) |  |
| **Adverse events (AEs)** |  |  |  |
| Any AE | 76 (58.4) | 7 (53.85) | 0.98 |
| Nausea (any grade) | 29 (22.3) | 5 (38.46) | 0.89 |
| Neutropenia (any grade) | 27 (20.7) | 3 (23.08) | 1 |
| Fatigue (any grade) | 42 (32.3) | 1 (7.69) | 0.38 |
| Dose reduction | 35 (26.9) | 2 (15.38) | 0.57 |
| Permanent discontinuation | 62 (47.6) | 6 (46.15) | 1 |
| **Toxicity grade** |  |  | 1 |
| G1/G2 | 106 (82) | 11 (84.62) |  |
| G3/G4 | 24 (18) | 2 (15.38) |  |
| **Drug-PIN score°** |  |  |  |
| Median (range) | 3 (1-18.4) | 3 (1.6-78.9) | 0.44 |
| **Drug-PIN light°** |  |  | 0.132 |
| Green | 116 (90) | 10 (76.92) |  |
| Yellow | 9 (7) | 2 (15.38) |  |
| Dark yellow | 3 (2.3) | 0 |  |
| Red | 1 (0.7) | 1 (7.69) |  |
| **Concomitant medications** |  |  | 0.27 |
| No | 76 (58.4) | 5 (38.46) |  |
| ≤3 | 47 (36.2) | 3 (23.08) |  |
| >3 | 7 (5.4) | 5 (38.46) |  |
| **Comorbidities** |  |  | 0.045 |
| No | 99 (76.15) | 6 (46) |  |
| Yes | 31 (23.85) | 7 ( 54) |  |

Legend N: number of patients; ER: estrogen receptor; T-DXd: trastuzumab deruxtecan; AE: adverse event; NA: not available * Percentage calculated among female patients only, ° Percentage calculated among evaluable patients (N=142). P refers to p-values obtained from ChiSquare or Fischer’s Exact test for categorical variables, and from Student’s t-test for continuous variable (i.e., drug PIN score).
